# Key clinical predictors in the diagnosis of ovarian torsion in children

**DOI:** 10.1016/j.jped.2024.01.006

**Published:** 2024-04-03

**Authors:** Sai Chen, Zhigang Gao, Yunzhong Qian, Qingjiang Chen

**Affiliations:** Zhejiang University School of Medicine, The Children's Hospital, National Clinical Research Center for Child Health, Department of Pediatric General Surgery, Zhejiang Province, China

**Keywords:** Ovarian torsion, Children, Immune cells, Prenatal examination, Precocious puberty

## Abstract

**Objective:**

Ovarian torsion (OT) represents a severe gynecological emergency in female pediatric patients, necessitating immediate surgical intervention to prevent ovarian ischemia and preserve fertility. Prompt diagnosis is, therefore, paramount. This retrospective study set out to assess the utility of combined clinical, ultrasound, and laboratory features in diagnosing OT.

**Methods:**

The authors included 326 female pediatric patients aged under 14 years who underwent surgical confirmation of OT over a five-year period. Logistic regression analysis was employed to pinpoint factors linked with OT, and the authors compared clinical presentation, laboratory results, and ultrasound characteristics between patients with OT (OT group) and without OT (N-OT group). The authors conducted receiver operating characteristic (ROC) curve analysis to gauge the predictive capacity of the combined features.

**Results:**

Among 326, OT was confirmed in 24.23 % (79 cases) of the patients. The OT group had a higher incidence of prenatal ovarian masses than the N-OT (22 cases versus 7 cases) (*p* < 0.0001). Similarly, the authors observed significant differences in the presence of lower abdominal pain, suspected torsion on transabdominal ultrasound, and a high neutrophil-lymphocyte ratio (NLR > 3) between the OT and non-OT groups (p ˂ 0.05). Furthermore, when these parameters were combined, the resulting area under the curve (AUC) was 0.868, demonstrating their potential utility in OT diagnosis.

**Conclusion:**

This study demonstrates a prediction model integrating clinical, laboratory, and ultrasound findings that can support the preoperative diagnosis of ovarian torsion, thereby enhancing diagnostic precision and improving patient management. Future prospective studies should concentrate on developing clinical predictive models for OT in pediatric patients.

## Introduction

Ovarian lesions, although rare in the pediatric population, display clinical features and pathology distinct from those of adults. They constitute 1 % to 5 % of all pediatric and adolescent cases, with some studies noting approximately 2.6 instances of ovarian complications per 100,000 female pediatrics.^1^ One particular concern is ovarian torsion (OT), an ovarian lesion that can affect females of all ages.^2^ The incidence of OT among children varies from 4.9/100,000 to 20-30/100,000, with an average age of 13 years.^3^

Ovarian torsion is a severe condition where the ligament support of the ovary or its appendages twist, obstructing the ovarian parenchymal veins and lymph nodes.^4^^,^^5^ If not diagnosed and treated promptly, it can escalate to congestion and hemorrhagic necrosis, arterial obstruction, and potentially ovarian necrosis. This sequence of events can further impact growth, development, reproduction, and endocrine function. The pathophysiology of OT involves various factors, such as changes in intra-abdominal pressure, tubal spasms, ligament overactivity, and hormonal activity during the premenstrual and peripartum periods.^3^^,^^5^

Diagnosing OT in children is complex, as it often presents with varying degrees of lower abdominal pain, nausea, vomiting, fever, and other symptoms that lack specificity. Previous research showed that only 38 % of preoperative diagnoses were correct, emphasizing the challenge of early detection.^6^ Furthermore, infantile OT presents more variability on ultrasound than in older children and adolescents due to the possibility of prenatal occurrence and asymptomatic presentation.^7^ As such, potential signs of OT detected during prenatal exams should be considered when diagnosing ovarian conditions in children.

Historically, blood tests and biomarkers, including interleukin-6 (IL-6), D-dimer, C-reactive protein (CRP), white blood cell (WBC) count, and neutrophil-lymphocyte ratio (NLR), have been used to assist OT diagnosis.^8^^,^^9^ These biomarkers change in response to the systemic inflammatory reaction induced by ovarian damage and necrosis from torsion. Of these, the NLR has been noted for its quick and accurate response to inflammatory changes, making it a significant inflammatory marker for various diseases.^10^^,^^11^

Despite the fact that ultrasound has been used as a useful diagnostic tool, its limitations, such as missed swirl signs or misleading Doppler flow patterns, have raised concerns over its sensitivity and accuracy.^12^, ^13^, ^14^ Therefore, there is a growing interest in incorporating additional clinical parameters and exploring alternative imaging techniques, such as computed tomography (CT) scans or magnetic resonance imaging (MRI), to enhance diagnostic precision.^15^^,^^16^

Moreover, research endeavors are focused on developing novel biomarkers or molecular signatures specific to OT. The discovery of such markers could revolutionize diagnostics, offering rapid and accurate detection before symptoms manifest or when traditional imaging techniques are inconclusive. While ultrasound remains a fundamental diagnostic tool due to its noninvasive nature and widespread availability, its use in conjunction with other diagnostic modalities is recommended for a comprehensive assessment. Collaborative efforts among clinicians and researchers are expected to lead to improved methodologies, enhancing our ability to promptly and accurately diagnose OT.

The importance of preoperative diagnosis of OT in medical practice is well recognized. Regardless of advancements in technology and surgical procedures, direct observation during surgery remains the gold standard for definitive diagnosis. Timely intervention and prevention of further complications are facilitated by accurate preoperative diagnosis, which is essential for ensuring favorable patient outcomes.^17^

Despite extensive research, OT diagnosis remains challenging. This paper aims to provide a comprehensive analysis of sonographic, clinical, and laboratory features of OT in children. By doing so, the authors hope to shed light on new perspectives that can enhance preoperative diagnosis and thereby improve patient care.

## Methods

### Study design and patient enrollment

This study employed a retrospective design approved by institutional review boards to evaluate clinical, ultrasound, and laboratory features for their potential in diagnosing ovarian torsion (OT) among female pediatric patients who presented to the emergency department. Between August 2017 and March 2022, the authors enrolled 326 female pediatric patients who required laparoscopy due to suspected ovarian disease. Post-laparoscopy, the authors categorized patients into the ovarian torsion (OT) group, consisting of 79 patients with ovarian torsion, and the non-ovarian torsion (N-OT) group, which included 247 patients without ovarian torsion.

Patients under 12 years of age and for whom complete abdominal ultrasound imaging data with good image quality was available were included in the study. The exclusion criteria were: (1) Premature infants, macrosomia infants, and low-weight birth infants; (2) Secondary ovarian torsion; (3) No abdominal ultrasound imaging examination was performed before surgery. (4) Patients younger than 1 year were excluded because of the high prevalence of fetal ovarian cysts in this age group; (5) The authors also excluded cases of prenatal ovarian torsion because the condition has significant differences in presentation and physical examination.

A flow chart of the study design is shown in Supplemental Fig. 1.

### Demographic and clinical data collection

An in-depth demographic study was conducted for all the participants, focusing on aspects such as age and the location of ovarian masses. Other clinical characteristics were also noted, such as body mass index, duration of pain, vomiting, lower abdominal pain, absence of leukorrhea and metrorrhagia, vaginal bleeding, history of ovarian cyst, and palpable abdominal mass as well as the incidence of ovarian masses detected before birth and the prevalence of precocious puberty.

### Sonographic and laboratory analysis

The authors undertook a comprehensive comparison of clinical symptoms, ultrasound features, and laboratory parameters between the OT and N-OT groups. The authors compared abdominal ultrasound examination i.e., transabdominal sonography (TAS), C-reactive protein level (CRP), white blood cell count, absolute neutrophil count (ANC), and NLR between two groups of patients to determine whether ovarian torsion occurred. Referring to relevant articles, different studies often use NLR > 3 as the threshold to analyze the relationship between NLR and diseases.^18^^,^^19^ Therefore, in the present study, the authors used the diagnostic value of NLR as NLR > 3 for predicting patients with OT.

The Siemens S2000 and Philips EPIQ5 color Doppler ultrasound diagnostic instruments were used for an abdominal ultrasound. Convex array probe, frequency 2–6 MHz; Linear array probe, frequency 5–12 MHz. All patients were placed in a supine position, and the pelvic cavity was scanned to find the ovaries. The bladder was filled as much as possible. Routine abdominal and pelvic examinations were performed, with a focus on scanning the uterus and bilateral appendages. The size of the uterus and bilateral ovaries was measured, and the morphology, structure, volume, internal echo, blood flow changes, and pelvic fluid accumulation of the bilateral ovaries were observed. At the same time, the appendix area was also scanned to exclude other acute abdominal conditions such as appendicitis. If the ultrasound imaging shows significant ovarian enlargement and is located in an uncommon area, with uneven echo, no blood flow signal in the ovary, and torsion of the ovarian vascular pedicle, it can be determined as ovarian torsion. The evaluation of abdominal ultrasound was performed by two board-certified radiologists.

### Statistical analysis

Descriptive statistics (mean ± SD for continuous data and frequencies for categorical data) were used to present the demographic and health aspects of the two groups. To compare clinical, sonographic, and laboratory variables between the OT and N-OT groups, the authors utilized the chi-square test or Fisher's exact test for categorical variables and the Mann-Whitney test for continuous variables. Variables that were associated with ovarian torsion in the multivariate analysis and were of clinical significance, including lower abdominal pain, CRP, prenatal detection of ovarian mass, NLR > 3, and suspected torsion by TAS, were incorporated into a logistic regression model. To refine this model, the authors employed backward selection to eliminate variables that didn't significantly contribute information, given the other factors in the model. After establishing the final model, the authors evaluated the diagnostic ability of a combination of prenatal examination of ovarian mass, suspected torsion by TAS, and NLR > 3 (referred to as the 'triad') to predict ovarian torsion. To determine the sensitivity, specificity, and cutoff values for the 'triad' and the presence of at least two of these features, the authors constructed a receiver operating characteristic (ROC) curve and calculated the area under this curve.

## Results

### Demographic and clinical characteristics

A total of 326 female pediatric patients were included in the study. Among them, approximately 24.23 % (79 cases) were diagnosed with ovarian torsion (OT), while the remaining 75.77 % (247 cases) had no ovarian torsion (N-OT). The ages of the participants in the OT group and N-OT group were 3155±1637 days and 3436 ± 1269 days, respectively. There was no significant difference in age between the two groups (*p* = 0.5761). Moreover, no significant differences were observed in the BMI, duration of pain, vomiting, lower abdominal pain, absence of leukorrhea and metrorrhagia, vaginal bleeding, and history of ovarian cyst between the two groups (*p* > 0.05) (Table 1).

**Table 1:** Demographic and clinical characteristics of patients who underwent laparoscopy.

Parameters	OT group	N-OT group	P-value
Cases (*N* = 326)	79	247	–
Age (d)	3155±1637	3436±1269	0.5761
Race/ethnicity (Han Chinese)	79 (100 %)	242 (97.9 %)	0.963
BMI (kg/m^2^) (main) ± SD	19.26±2.32	18.90±1.92	0.174
Duration of pain (d)(mean) ± SD	5.59±1.48	5.40±1.63	0.337
Vomiting (N) (%)	6 (7.59 %)	13 (5.26 %)	0.4184
Absence of leukorrhea & metrorrhagia	75 (94.9 %)	247 (100 %)	0.23
Vaginal bleeding	6 (7.6 %)	25 (10.1 %)	0.77
History of ovarian cyst	0 (0.0 %)	1 (2.6 %)	1.00
PE N (%)	22 (27.85 %)	7 (2.83 %)	<0.0001
PP N (%)	5 (6.33 %)	72 (29.15 %)	<0.0001

BMI, Body-mass index; PE, Prenatal examination revealed ovarian mass; PP, Precocious puberty.

### Prevalence of ovarian masses and precocious puberty

The study revealed a significant disparity in the occurrence of ovarian masses detected before birth between the OT and N-OT groups. The OT group had a higher incidence of such masses than the N-OT (22 cases versus 7 cases, respectively), and statistical analysis confirmed this discrepancy as highly significant (*p* < 0.0001) (Table 1).

Moreover, there was a significant difference in the prevalence of precocious puberty between the two groups. The OT group had lower rates of early-onset puberty than the N-OT group (5 cases versus 72 cases, respectively), and statistical analysis again confirmed these results as highly significant (*p* < 0.0001) (Table 1).

### Clinical, sonographic and laboratory parameters

Table 2 presents the comparison of clinical presentation, ultrasound features, and laboratory parameters between the OT and N-OT groups. The OT group had a higher proportion of children with lower abdominal pain (55.67% vs. 35.22 %, *p* < 0.001) and a higher proportion of children with suspected torsion according to the TAS (54.53% vs. 10.5 %, *p* < 0.001). There were more patients in the OT group with lower abdominal pain than in the N-OT group and the difference was significant (*p* < 0.001). Additionally, the OT group had significantly higher levels of CRP, WBC count, and ANC (*p* < 0.001). The NLR was also higher in the OT group, with 41.77 % of patients in the OT group having an NLR > 3 compared to only 3.64 % in the N-OT group (*p* < 0.001) (Table 2). Moreover, the authors found that NLR > 3 had a sensitivity of 82.3 % and a specificity of 85 % for predicting ovarian torsion (Supplemental Fig. 2).

**Table 2:** Clinical, sonographic, and laboratory findings at presentation.

Indicators	OT group	N-OT group	p-value
Palpable abdominal mass (N) (%)	5 (3.95 %)	77 (31.17 %)	<0.0001
Lower abdominal pain (N) (%)	44 (55.67 %)	87 (35.22 %)	**<**0.0001
Fever (N) (%)	7 (8.8 %)	0 (0.0 %)	0.34
Ovarian torsion (Left) (N)	29	104	0.396
Ovarian torsion (Right) (N)	50	143	
WBC (*10^9/L) (main) ± SD	10.73±3.83	7.649±2.965	<0.0001
Absolute Lymphocytes (*10^9/L) (mean) ± SD	3.368±2.321	2.696±1.174	0.2949
Percentage of Lymphocytes (mean) ± SD	34.25±22.06	37.49±14.54	0.0222
Absolute Neutrophils (*10^9/L) (mean) ± SD	6.561±4.354	4.283±2.645	<0.0001
Percentage of Neutrophils (mean) ± SD	57.55±24.04	53.55±15.85	0.0143
CRP (mg/L) (mean) ± SD	5.891±14.71	1.823±7.838	<0.0001
NLR (mean) ± SD	3.769±4.475	0.9171±0.9147	<0.0001
NLR > 3 (N) (%)	33 (41.77 %)	9 (3.64 %)	<0.0001

WBC, White Blood Cells; CRP, C-Reactive Protein; NLR, Neutrophil -Lymphocyte Ratio.

### Predictors of ovarian torsion

A multivariate analysis was performed for different factors associated with ovarian torsion. The odds ratios for different factors associated with ovarian torsion are presented in Supplemental Table 1. Patients with suspected torsion identified by TAS were 9.17 times more likely to have adnexal torsion than those without (95 % CI: 4.434–18.965). Notably, patients with NLR > 3 had a higher incidence of ovarian torsion than those with NLR < 3, and the odds ratio was 10.847 (95 % CI: 4.283–27.474). In addition, patients with ovarian masses detected by prenatal examination were 20.377 times more likely to have adnexal torsion than those without (95 % CI: 7.295–56.915).

### Predictive value of triad and combination features

Prenatal examination of the ovarian mass, suspected torsion by TAS, and NLR > 3 were combined as a triad. Table 3, Table 4 show the sensitivity, specificity, cutoff values, and area under the curve (AUC) for the triad and combinations of at least two features. When all features of the triad were present, the probability of predicting ovarian torsion had a sensitivity of 82.3 %, a specificity of 85 %, a cutoff value of 0.673, and an AUC of 0.868. In the presence of at least two features, sensitivity ranged from 65.8 % to 82.3 %, and specificity ranged from 85 % to 87 %, depending on the combination of signs.

**Table 3:** The role of various combinations of the triad of factors (TAS, NLR > 3, and PE) in predicting adnexal torsion.

Signs of torsion	TAS	NLR >3	PE	Predicted probability for torsion
0 of 3	−	−	−	0.07009
1 of 3	−	+	−	0.44983
	−	−	+	0.60567
	+	−	−	0.40871
2 of 3	+	+	−	0.88232
	+	−	+	0.93371
	−	+	+	0.94338
3 of 3	+	+	+	0.99350

TAS, Transabdominal ultrasound; NLR, Neutrophil-lymphocyte Ratio: PE, Prenatal examination revealed ovarian mass.

**Table 4:** The sensitivity, specificity, cutoff values, and AUC for the triad.

Indicators	Sensitivity	Specificity	Cutoff values	AUC	95 % CI
TAS+NLR	0.658	0.862	0.52	0.782	.0714–0.849
TAS+PE	0.747	0.87	0.617	0.818	0.757–0.879
NLR+PE	0.649	0.951	0.597	0.795	0.728–0.861
TAS+NLR+PE	0.823	0.85	0.673	0.868	0.814–0.922

TAS, Transabdominal ultrasound; NLR, Neutrophil-lymphocyte Ratio; PE, Prenatal examination revealed ovarian mass.

## Discussion

Childhood ovarian torsion studies are relatively scarce, as the current literature primarily focuses on adult cases. Ovarian torsion in pediatric patients predominantly originates from congenital or physiological factors such as hormonal changes causing ovary enlargement or ligament elongation. Given children's long life expectancy, the risks posed by ovarian torsion are especially pronounced, potentially leading to severe complications such as ovarian necrosis and peritonitis. Therefore, it is critical to diagnose this condition early and correctly to enhance ovarian salvage rates, which currently range from 27 % to 99 %.^20^

The present study involved 326 children who underwent laparoscopic surgery for ovarian masses, out of whom 79 had ovarian torsion. The key predictors of adnexal torsion were suspected torsion via abdominal ultrasound, and prenatal examination revealing an ovarian mass, with NLR ˃ 3. Ultrasonography revealed multiple follicles surrounding the enlarged ovaries, as well as abnormal blood flow signals, indicating a series of ovarian torsion sonograms. However, as ultrasound results hinge on the examiner's skill and experience, disparities inevitably arise, exposing a drawback of this diagnostic method. Hence, there is a need for a collective diagnosis considering prenatal ovarian mass detection, NLR > 3, and ultrasound results. Combining these three parameters for diagnosing and predicting ovarian torsion resulted in a low sensitivity yet an impressive specificity of 95.1 %. When at least two of these features were present, this model predicted torsion with a probability ranging from 88.23 % to 99.35 %. The present research concurs with other studies reporting that approximately 40 % of surgically confirmed ovarian torsion cases were preoperatively diagnosed based on clinical and ultrasound characteristics.^21^^,^^22^ Despite the reliance on TAS for ovarian torsion diagnosis, the authors observed a low sensitivity of 54.4 % for TAS alone. This corresponds with other findings revealing normal ultrasounds in approximately 50 % of ovarian torsion cases.^23^ These discrepancies challenge the exclusive reliance on ultrasound for preoperative diagnosis.

Further analysis of the present data demonstrated that lower abdominal pain was significantly more common in patients with ovarian torsion than in those without (55.67% vs. 35.22 %, *p* < 0.001). This aligns with previous findings linking lower abdominal pain and adnexal torsion.^24^^,^^25^ Likewise, several studies show the same symptom as a potential indicator of gynecological inflammation and malignancies.^8^^,^^24^^,^^26^

The present research also established a significantly higher NLR in patients with ovarian torsion than in those without (3.769 ± 4.475 vs. 0.9171 ± 0.9147, *p* < 0.001), confirming prior research.^18^^,^^27^ The rise in NLR stems from the inflammatory and immune response triggered by ischemia and necrosis in ovarian torsion, making it a potential early diagnostic and assessment tool for this condition.

Currently, no universally accepted predictive rule exists for adnexal torsion detection. Given the insufficiency of any single tool for a reliable diagnosis, the authors propose a simple prediction model for ovarian torsion based on symptoms and laboratory and ultrasound findings. The authors report a specificity of 95.1 % and an AUC of 0.795 for the combination of NLR > 3, prenatal examination revealing ovarian mass, and suspected torsion on abdominal ultrasound. Despite its low sensitivity, the model's high specificity indicates its importance. Thus, the presence of at least two of these three features should heighten the clinical suspicion of ovarian torsion in children with ovarian masses.

### Strengths and limitations

This study possesses a number of notable strengths. To our knowledge, this is the first study to investigate the clinical symptoms, NLR, and ultrasound characteristics of pediatric ovarian torsion both independently and in combination. The study is further distinguished by its substantial sample size, constituting the largest single-institutional cohort of children with torsion diagnosed via laparoscopic findings. Moreover, the urgency and real-world applicability of the present research are emphasized by the fact that most abdominal sonograms were carried out in emergency room settings, reflecting the critical need for timely diagnosis of suspected adnexal torsion.

However, the authors recognize several limitations inherent in the research design. Primarily, the retrospective nature of the study could introduce bias, potentially affecting the results. Furthermore, the authors did not comprehensively document the ultrasound features of adnexal torsion, limiting descriptions to a binary categorization of torsion suspected or not suspected. The present sample is also exclusively composed of female pediatric patients who underwent surgical treatment, possibly excluding cases of adnexal torsion that did not receive surgery. However, given the status as a regional national medical center, the authors often receive referrals from other institutions, which likely reduces the number of missed cases.

Despite these limitations, the present findings underscore the importance of ultrasound, clinical symptoms, and laboratory features as first-line evaluative tools for children suspected of having ovarian torsion. The presence of an ovarian mass upon prenatal examination, an NLR > 3, and indicative findings from TAS should raise a high suspicion of torsion.

In light of these findings, the authors developed a predictive model incorporating these three features to aid in the preoperative diagnosis of torsion in children. This tool can expedite diagnosis, enabling prompt surgical intervention and potentially mitigating the risk of ovarian damage.

Nevertheless, the retrospective design of this study restricts the ability to draw definitive conclusions. As such, further prospective studies are warranted to corroborate the present findings and refine the predictive model. As medical professionals, the goal is to continually improve diagnostic capabilities to increase the chances of preserving ovarian function and improving patient outcomes.

## Conclusions

This study presents a prediction model that combines clinical, laboratory, and ultrasound findings to assist in the preoperative diagnosis of children with ovarian torsion, thus proving its usefulness in the emergency department.

## Conflicts of interest

The authors declare no conflicts of interest.

## Appendix Supplementary materials

Image, application 1
