# Supplementary material for: Key clinical predictors in the diagnosis of ovarian torsion in children
Source: J Pediatr (Rio J). 2024 Apr 3;100(4):399–405. doi: 10.1016/j.jped.2024.01.006 (PMC11331230; doi:10.1016/j.jped.2024.01.006)
Supplement: Supplementary file 1 [file mmc1.docx]

**JPED-D-23-00325_Supplemental material**

**
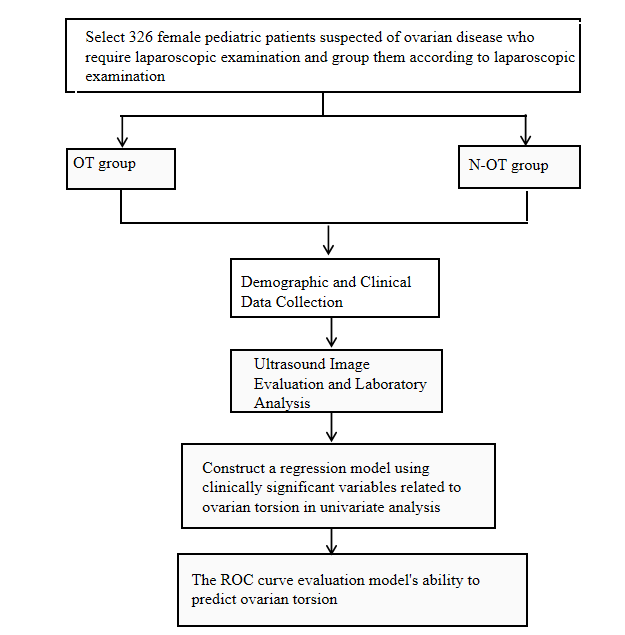
**

**Supplemental Figure 1.** The flow chart of the study design.


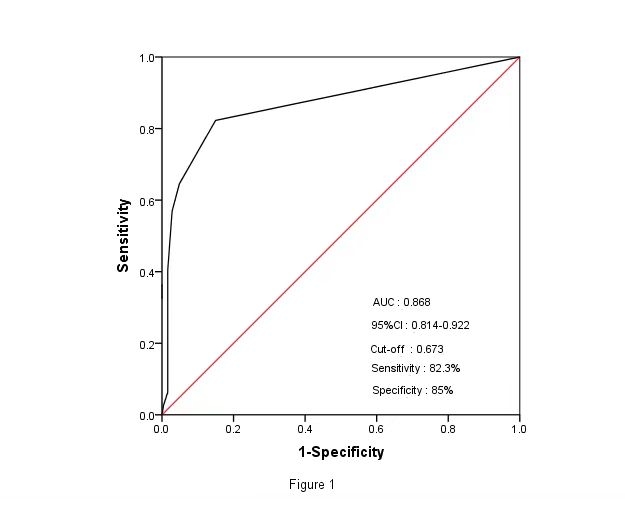


**Supplemental Figure** **2.** Receiver operating characteristic (ROC) curve of Neutrophil-Lymphocyte Ratio (NLR>3) in ovarian torsion (OT) patients. ROC curve analysis for NLR >3 in the diagnosis of OT. NLR >3 had a sensitivity of 82.3% and a specificity of 85%.

**Supplemental Table 1.** Multivariate analysis of factors associated with Ovarian torsion.

| **Indicators** | **OR** | **95% CI** |
| --- | --- | --- |
| TAS | 9.17 | 4.434-18.965 |
| NLR | 10.847 | 4.283-27.474 |
| PE | 20.377 | 7.295-56.915 |

TAS, Transabdominal ultrasound; NLR, Neutrophil-lymphocyte Ratio; PE, Prenatal examination revealed ovarian mass.
